# Male vocalizations convey information on kinship and inbreeding in a lekking bird

**DOI:** 10.1002/ece3.4986

**Published:** 2019-03-28

**Authors:** Clément Cornec, Alexandre Robert, Fanny Rybak, Yves Hingrat

**Affiliations:** ^1^ Institut des Neurosciences Paris‐Saclay Université Paris‐Sud, CNRS (UMR 9197) Orsay France; ^2^ Emirates Center for Wildlife Propagation Missour Morocco; ^3^ Centre d'Ecologie et des Sciences de la Conservation (CESCO), Muséum national d'Histoire naturelle, Centre National de la Recherche Scientifique Sorbonne‐Université Paris France; ^4^ Reneco International Wildlife Consultants LLC Abu Dhabi UAE

**Keywords:** acoustic signal, *Chlamydotis undulata undulata*, exploded lek, inbreeding, kinship

## Abstract

Kinship and inbreeding are two major components involved in sexual selection and mating system evolution. However, the mechanisms underlying recognition and discrimination of genetically related or inbred individuals remain unclear. We investigated whether kinship and inbreeding information is related to low‐frequency vocalizations, “booms,” produced by males during their courtship in the lekking houbara bustard (*Chlamydotis undulata undulata*). Based on a captive breeding program where the pedigree of all males is known, we investigated the similarity of booms’ acoustic parameters among captive males more or less individually inbred and therefore genetically related with each other. In the wild, we investigated the relationship between the spatial distribution of males within leks and the similarity of acoustic parameters of their booms. In the captive population, we found (a) a relationship between the individual inbreeding level of captive males and their vocalization parameters; (b) that kin share similar frequency and temporal characteristics of their vocalizations. In the wild, we found no evidence for spatial structuring of males based on their acoustic parameters, in agreement with previous genetic findings on the absence of kin association within houbara bustard leks. Overall, our results indicate that genetic information potentially related to both the identity and quality of males is contained in their vocalizations.

## 1 INTRODUCTION

In the context of sexual selection, one major element of the mating system and reproductive behavior is the communication between individuals, allowing the attraction and stimulation between potential sexual partners, as well as interactions between individuals of the same sex (Andersson, 1994). Visual, electric, acoustic, and chemical signals (hereafter, signals) may convey several levels of information on breeders such as quality (of fitness‐related traits), motivational, and receptivity states, as well as identity. Identity signals, allowing distinguishing conspecifics, groups, individuals, and even kin, are of crucial importance to reproductive success and are likely to be the target of sexual selection (Bradbury & Vehrencamp, 2011).

The ability to distinguish kin from nonkin is an important driving force in the evolution of social and sexual behaviors (Penn & Frommen, 2010) and appears to be particularly relevant in species with a lek mating system (Kokko & Lindström, 1996; Sherman, 1999). Within leks, sexual selection is intense because females attend male aggregates primarily for mating, and male reproductive investment is limited to the fertilization of female gametes, leading to high level of competition between males (Höglund & Alatalo, 1995). In such context, males with low mating probability might preferentially join leks where the dominant male is a close relative, through kin recognition and association (kin selection; Kokko & Lindström, 1996). Larger leks being more attractive for females (Höglund & Alatalo, 1995), their increase in size through the aggregation of low‐ranked males with higher ranked kin, provides direct (increase per capita copulations) and indirect (inclusive fitness) benefits for both male categories (Sæther, 2002). Kin clustering has been shown in several lekking species, as peafowls *Pavo cristatus* (Petrie, Krupa, & Burke, 1999), prairie‐chickens *Tympanuchus pallidicentus* (Bouzat & Johnson, 2004), white‐bearded manakins *Manacus manacus* (Höglund & Shorey, 2003; Shorey, Piertney, Stone, & Höglund, 2000), and wild turkeys *Meleagris gallopavo* (Krakauer, 2005). However, many other studies failed in finding kin clustering within leks (Gibson, Pires, Delaney, & Wayne, 2005; Loiselle et al., 2006; McDonald, 2009; for review: Lebigre, Alatalo, Soulsbury, Höglund, & Siitari, 2014). Interestingly, most previous studies in lekking species investigated kin clustering in a cooperative perspective and as a possible explanation of lek formation (Kokko & Lindström, 1996). Nevertheless, kin recognition does not always lead to kin association (Penn & Frommen, 2010) and the absence of clusters of related males might be the result of kin competition and active kin avoidance (West, Pen, & Griffin, 2002). Whereas the absence of between‐sex kin association has been widely documented in relation to mate choice as an important mechanism of inbreeding avoidance (Bonadonna & Sanz‐Aguilar, 2012; Reynolds et al., 2014), its importance in shaping within sex interactions has received little attention in birds (but see Hardouin, Legagneux, Hingrat, & Robert, 2015).

Despite the empirical support to the existence of kin recognition in several lekking species, mechanisms and cues involved in the process per se remain largely enigmatic. Several studies have shown that signals may convey information on the coefficient of kinship (hereafter, kinship) between two individuals (see Kempenaers, 2007). Most traits associated with identity or quality are to some degree heritable through genetic transmission to the progeny as shown in a variety of taxa and for multiple sensory channels (e.g., odor: ring‐tailed lemur *Lemur catta*, Charpentier, Boulet, & Drea, 2008; acoustic signals: zebra finch *Taeniopygia guttata*, Forstmeier, Burger, Temnow, & Derégnaucourt, 2009; visual traits: guppies *Poecilia reticulate*; Houde, 1992). The heritability of these traits confers convergent signal profiles among related individuals compared to unrelated individuals. Therefore, individuals can use these traits to assess their degree of relatedness with conspecifics to distinguish kin and in turn adapt their behavioral response in terms of cooperation or competition (Bonadonna & Sanz‐Aguilar, 2012; Hamilton 1964a, 1964b; Hesse, Bakker, Baldauf, & Thünken, 2012; Parr, Heintz, & Wroblewski, 2010; Waldman, Rice, & Honeycutt, 1992). Contrary to what is observed in cooperatively breeding species, males of lekking species take no part in reproduction after mating and therefore offspring cannot learn the identity of their father. Petrie et al. (1999) showed that in spite of the absence of any previous period of association, genetically related peacocks are effectively associated within the lek, suggesting the possibility of self‐referent phenotype matching through heritable phenotype cues (Holmes & Sherman, 1982). Vocalizations may be good candidates to support such kinship information as acoustic parameters partly reflect heritable morphological, physiological, and neurological traits involved in the sound production mechanisms (e.g., Gee, 2005; Forstmeier et al., 2009). However, in lekking species, the heritability of vocal traits and their implications in kin discrimination remains to be demonstrated.

In addition to carry information on kinship, secondary sexual traits may also reflect intrinsic, individual genetic characteristics. For example, courtship behavior has been shown to be influenced by the individual inbreeding coefficient (equal to the kinship of the parents of the focal individual, hereafter, “inbreeding”: Ahtiainen, Alatalo, Mappes, & Vertainen, 2004; Aparicio, Cordero, & Veiga, 2001; Charpentier, Drea, & Williams, 2008; Charpentier, Drea et al., 2008; Foerster, Delhey, Johnsen, Lifjeld, & Kempenaers, 2003; Reid et al., 2005; Seddon, Amos, Mulder, & Tobias, 2004). In turn, courtship traits potentially influence female mating choice, with inbred males having a reduced mating success (Höglund et al., 2002; Ryder, Tori, Blake, Loiselle, & Parker, 2010). Similarly, traits associated with the degree of inbreeding may also play an important role in the agonistic relationship between males. Inbred males may develop weak expression of sexual, morphological, and behavioral traits, thereby giving them low competitive ability compared to outbred males (Hoffman, Forcada, Trathan, & Amos, 2007; Höglund et al., 2002; Ryder et al., 2010). However, in spite of its substantial importance in intra‐ and intersexual interactions, the relationship between individual inbreeding, inbreeding depression, and the elaboration of secondary sexual traits has been poorly investigated in bird species with a lek mating system.

In the lekking North‐African houbara bustard (*Chlamydotis undulata undulata*, hereafter houbara), the natal dispersal distance in males is of limited range (35 ± 20 km; Hardouin et al., 2012), and, once established, males remain faithful to their displaying site for years (Hingrat, Saint Jalme, Chalah, Orhant, & Lacroix, 2008). Consequently, dispersing males are likely to encounter male kin when establishing in a lek. However, an analysis of relatedness between 73 males from 11 leks in the same region indicated an absence of kin association within leks (Lesobre, 2008). This suggests a possible kin recognition process followed by an active kin avoidance when males establish in leks. In the houbara, acoustic traits (booming calls) are strong components of the courtship and convey information on male quality, with booms of lower frequency indicating males of better quality in terms of male health status and breeding success (Chargé, Saint Jalme, Lacroix, Cadet, & Sorci, 2010; Cornec, Hingrat, Robert, & Rybak, 2015). In the present study, by taking advantage of a conservation breeding, where the pedigree of all captive males is known (Chargé et al., 2014), we investigated acoustic parameters in captivity in related males to test whether kinship information is signaled in the vocalizations produced during the courtship. We also tested the relationship between the vocalization of males and their inbreeding coefficient. In parallel, we investigated in the wild the spatial distribution of displaying males of various leks with regard to the similarity of their vocalizations. With respect to our analyses on captive males, we predicted that (a) there will be a positive relationship between kinship and vocalization parameters, and (b) the most inbred males will produce higher frequency booms indicating lower quality (Cornec et al., 2015).

Owing to the relatively small size of our sample of males in the wild, we did not make formal predictions regarding the relationship between the spatial distribution of these males and the similarities of their vocalizations. However, because previous findings indicate an absence of kin association among males within leks (Lesobre, 2008), we did not expect any strong association between spatial location and vocalization parameters.

## 2 MATERIAL AND METHODS

### 2.1 Study in captivity

#### 2.1.1 Breeding program

The study was carried out in the conservation breeding of the Emirates Center for Wildlife Propagation (www.ecwp.org) in Morocco. Birds are housed in outdoor individual cages (2 × 2 × 2 m) arranged in rows. Males can be in visual and acoustic contact with each other but are separated from females. Reproduction is achieved by artificial insemination (Saint Jalme, Gaucher, & Paillat, 1994). The breeding program aims at equalizing the founders’ contribution and maximizing the genetic diversity (Lesobre, 2008). All captive birds have a known and available pedigree (see Chargé et al., 2014).

#### 2.1.2 Subjects

The study was conducted on 36 captive males with contrasted kinship relationships, from March 14, 2013 to April 26, 2013. All the males used were between 4 and 13 years old and sexually mature, that is, providing viable semen used for insemination. We recorded nine groups composed of closely related males, each group including one sire and his sons. The nine sires were aged from 9 to 13 years, and their sons (18 males, one to three per sire) were aged from 4 to 10 years. Additionally, to these nine groups of related males, nine nonrelated males aged from 4 to 10 years were recorded.

#### 2.1.3 Kinship and inbreeding coefficients calculation

Kinship and inbreeding coefficients were calculated using available pedigree information on the captive population. In general, the same male was used to inseminate a given female during a breeding season; however, when semen collection failed, the ejaculate of another male was used, incurring potential doubts about paternity. Reliability of pedigree structure was thus reinforced by performing microsatellite analyses to identify the sire (Lesobre, 2008). Kinship coefficients of all pairs of individuals considered in our sample (*N* = 36 individuals) and individual inbreeding coefficients were calculated using PEDIG software (Boichard, 2002). The inbreeding coefficient of an individual is defined as the probability that the two alleles in one locus are identical by descent. Inbreeding coefficients were calculated using the Cholesky factors of the relationship matrix (see details in Boichard, 2002; Meuwissen & Luo, 1992) as implemented in program *meuw.f *of PEDIG. The kinship coefficient is a measure of relatedness that represents the probability that two alleles at the same locus, one sampled at random from each individual, are identical by descent. Kinship coefficients were computed, in order to obtain a kinship coefficient matrix, by generating a “progeny” for each pair of sampled individuals and computing its inbreeding coefficient with Meuwissen's method (Meuwissen & Luo, 1992) as implemented in program *par3.f *of PEDIG.

#### 2.1.4 Acoustic recordings

At least ten booms with a sufficient signal‐to‐noise ratio (on average 13.8 ± 1.92 sequences of booms per male, corresponding to a range of 10 to 15 sequences of booms per male) to achieve further acoustic analyses were recorded per male. Recordings were performed using a microphone Gras 46AE (frequency response: 3.15 Hz–20 kHz ± 2 dB), connected to a Marantz PMD661 recorder (Frequency response: 20–24 kHz ± 1 dB, sampling frequency: 44.1 kHz). The microphone was positioned at the height of the male using a tripod and placed in the front of the cage (maximum 1 m). None of the recorded males showed any aversion to the material and all exhibited normal courtship behavior. The experimenter stood still at about 20 m from the cage so as to not disturb the courtship activity of the focal male and scored the times when the male produced booms, the general behavior of the male and all disturbances that occurred during recording. The recordings were made during intensity peaks of the display activity, that is, three hours after sunrise and/or three hours before sunset, and only when the weather condition was optimal (absence of rain or wind). Although males were recorded on the same day whenever it was possible, some were recorded over several days (maximum 4 days).

### 2.2 Study in the wild

#### 2.2.1 Study area and subjects

Recordings were carried out in 2010 and 2011 in Al Baten study area located in the Middle Atlas in eastern Morocco near to the town of Missour. Al Baten is a slightly undulating gravel plain (altitude from 800 to 1,700 m) of 663 km^2^ covered by sparse shrubby vegetation, that is steppe‐like formation. The climate is Mediterranean subdesertic with <200 mm of precipitation per year. A breeding population of Houbara Bustard protected from hunting since 1996 is present in the Al Baten region (Lacroix, 2003). Since 2002, display sites where males perform courtship during each breeding season are located every year (Hingrat et al., 2008, 2004). A total of 99 and 74 display sites were identified in 2010 and 2011, respectively. Enough sequences of booms with a sufficient signal‐to‐noise ratio (252 sequences of booms in total, corresponding to 13 to 15 sequences of booms per male) were successfully recorded from 9 males in 2010 and from 8 additional other males in 2011, aggregated in leks of at least 4 males.

#### 2.2.2 Spatial distance

The location of the display site of each recorded male was recorded on the field in decimal degrees using a GPS (GPSmap 276C, Garmin, accuracy <15 m). Coordinates were then projected under GIS software (Esri ArcGis; coordinate system UTM zone 30N) in order to obtain coordinates in UTM metric unity. Euclidean distances between points of display sites were then calculated in order to obtain a spatial distance matrix using Access software with the following formula:

$$\text{Euclidean distance}=\surd \{{\left(Xa-Xb\right)}^{2}+{\left(Ya-Yb\right)}^{2}\}$$

where *X* and *Y* are UTM projected coordinates in meters of points A and B.

#### 2.2.3 Acoustic recordings

Male vocalizations were recorded using a recording kit composed of a directional microphone Gras 46AE (frequency response: 3.15 Hz–20 kHz ± 2 dB), connected to a Marantz PMD670 recorder (Frequency response: 0–20 kHz ± 0.5 dB, sampling frequency: 22.05 kHz) powered by a 12 V battery, which was hidden in a dummy rock. Displaying males are highly mobile on their display sites, especially during the running phase, and the exact location where they stop to perform their booms is unpredictable. Thus, to maximize the chances of recording each male, three recording kits were installed three hours before sunset at the periphery of its display site (Cornec, Hingrat, & Rybak, 2014). The recording was run continuously from three hours before sunset to three hours after sunrise. No recording was carried out when weather conditions were not optimal (i.e., presence of rain or wind).

#### 2.2.4 Acoustic analysis

Booms were analyzed using Avisoft‐SASlab Pro (R.Specht, version 4.40; Avisoft Bioacoustics, Berlin, Germany). Prior to analysis, audio files were down‐sampled at Fe = 2,756 Hz and filtered to remove background noise (band pass: 30–400 Hz). Boom duration (BD) was measured on the waveform as well as the following frequency parameters measured on the frequency spectrum: the fundamental and harmonic frequencies (F0 and H1, H2), the frequencies corresponding to 25%, 50%, and 75% of the energy (quartiles Q25, Q50, Q75), and the value of energy below 100 Hz (*E* < 100 Hz). The frequency modulation (FM) was also assessed on the spectrogram by measuring the 1st harmonic at the start (H1s) and at the end (H1e) of the boom, estimating the duration between these two measurements, and calculating the slope using the equation FM = ((H1s–H1e)/duration)).

### 2.3 Statistical analysis

#### 2.3.1 Repeatability of the 11 acoustic parameters

We analyzed the repeatability of each acoustic parameter of the 36 captive males and its confidence interval based on 1,000 parametric bootstraps as implemented in the rptR package of R (Stoffel, Nakagawa, & Schielzeth, 2017). The statistical significance of the repeatability of each parameter was tested by a likelihood ratio test comparing the model fit of a model including the male ID random grouping factor and one excluding it.

#### 2.3.2 Discriminant function analysis

The measured acoustic parameters were standardized and included in a discriminant function analysis while including male ID as a random factor (DFA; Fisher, 1936) to reduce the number of dimensions based on linear combinations of acoustic variables. The two first discriminant functions (λ1 and λ2) which explained most of the variation were used as integrative descriptors of the acoustic parameters of the booms. We assessed the relative position of each male in the dimension system defined by the discriminant functions. The pair‐wise Euclidean distances between barycenters of scatterplots of each male were calculated in order to obtain an acoustic distance matrix between captive males. All analyses were performed using Statistica 6.1 (Statsoft, 2001).

#### 2.3.3 Relationship between acoustic and genetic distances

Mantel tests (Mantel, 1967) were used to test the statistical significance of matrix correlations based on pair‐wise distances, between the acoustic distance matrix and the genetic distance matrix obtained for captive males (i.e., one minus the kinship coefficient between each pair of males), and between the acoustic distance matrix and the spatial distance matrix obtained for wild males. The randomization Mantel test is interpreted as a Pearson correlation coefficient varying between −1 and 1 and tested by means of a randomization procedure. In our case, to calculate significance, the data were subjected to Monte Carlo randomization tests (9,999 randomized runs) where one of the matrices was held constant and the other had its rows and corresponding columns randomly permuted.

#### 2.3.4 Relationship between individual inbreeding and acoustic parameters

We assessed the relationship between individual inbreeding and acoustic parameters using mixed effects linear models, using R packages lme4 (Bates, Maechler, Bolker, & Walker, 2015), Effects (Fox, 2003) and lmerTest (Kuznetsova, Brockhoff, & Christensen, 2017) implemented in R 3.0.0 (R Development Core Team, 2013). Two distinct models were used with the discriminant functions scores of the two first discriminant functions identified by the DFA (λ1 and λ2) as dependent variables, with the inbreeding coefficient as the fixed effect independent variable and male ID as a random factor. We also provide separate analysis of the relationship between individual inbreeding and each of the 11 acoustic parameters using the same modeling approach.

## 3 RESULTS

### 3.1 Relationship between acoustic parameters and kinship in captive males

Among captive males, repeatabilities of acoustic parameters were generally high (range 0.34–0.90) and all differed significantly from zero (see detailed results in Supporting Information Table S1).

The DFA including the 11 parameters measured on booms of captive males identified 11 significant linear functions that allowed maximizing individual segregation. The DFA assigned 79.08% of the booms to the correct male and the cumulative percentage of variance explained by the first two functions was of 77.55% (lambda Wilks = 0.0000464, *F*(385.458) = 18.822, *p* < 0.001; Table 1). The acoustic parameters of each male were then summarized by the two single values of the first two discriminant functions values of the DFA (λ1 and λ2).

**Table 1:** The first two discriminant functions of the discriminant function analysis performed with 11 acoustic parameters of the booms produced by captive males (explaining 77.55% of the total variance)

Axis	λ1	λ2
BD	0.078	**−0.454**
Q25	−0.011	−0.082
Q50	−0.420	0.013
Q75	0.019	0.068
*E* < 100 Hz	**0.525**	0.040
F0	**−0.550**	−0.096
H1	**−0.793**	−0.080
H2	−0.346	0.039
H1s	−0.361	0.312
H1e	−0.496	−0.079
FM	0.001	**−0.735**
PEV (%)	47.158	30.392
RD (%)	79.079	

Note

PEV: proportion of variance explained; RD: discrimination rate.

Measures that contributed the most to the two first functions are in bold. Abbreviations and definitions of parameters are provided in the Section 2.2.4.

**Figure 1:**
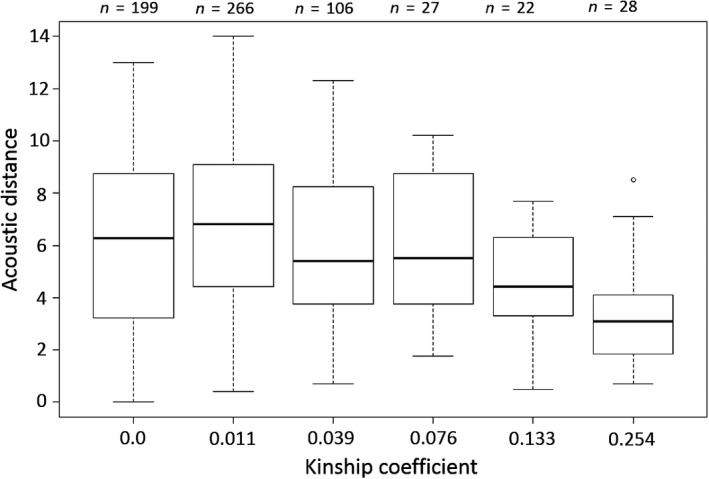
Relationship between acoustic and genetic distances in 36 houbara bustard captive males. Values on the *x*‐axis correspond to the mean kinship value for each kinship class. Numbers on top of the figure show sample sizes. The horizontal line that divides the box into two parts represents the median of the data. The end of the box shows the upper and lower quartiles. The whiskers show the highest and lowest value excluding outliers. Outliers are represented by open circles

The matrix of overall acoustic distances between males and the matrix of genetic distances between males were significantly correlated (simple Mantel test: *r* = 0.206, *p* < 0.001) (Figure 1): males whose booms were the most acoustically close were also the most related males.

The matrix of distances between mean λ1 of each male was significantly correlated with the genetic distance matrix between males (simple Mantel test: *r* = 0.142, *p* = 0.0015). The λ1 discriminant function, explaining 47% of the total variance was strongly correlated with F0, H1 and *E* < 100 Hz, thus the most related males had the most similar frequency parameters (Table 1). A significant correlation also existed between the matrix of distances between mean λ2 of each male and the genetic distance matrix (simple Mantel test: *r* = 0.148, *p* = 0.0033). The second discriminant function λ2 was strongly correlated with boom duration (BD) and frequency modulation (FM) and explained 30% of the total variance. Thus, the most related males produced booms with the closest duration and the most similar FM slope (Table 1).

### 3.2 Relationship between acoustic parameters and inbreeding in captive males

**Figure 2:**
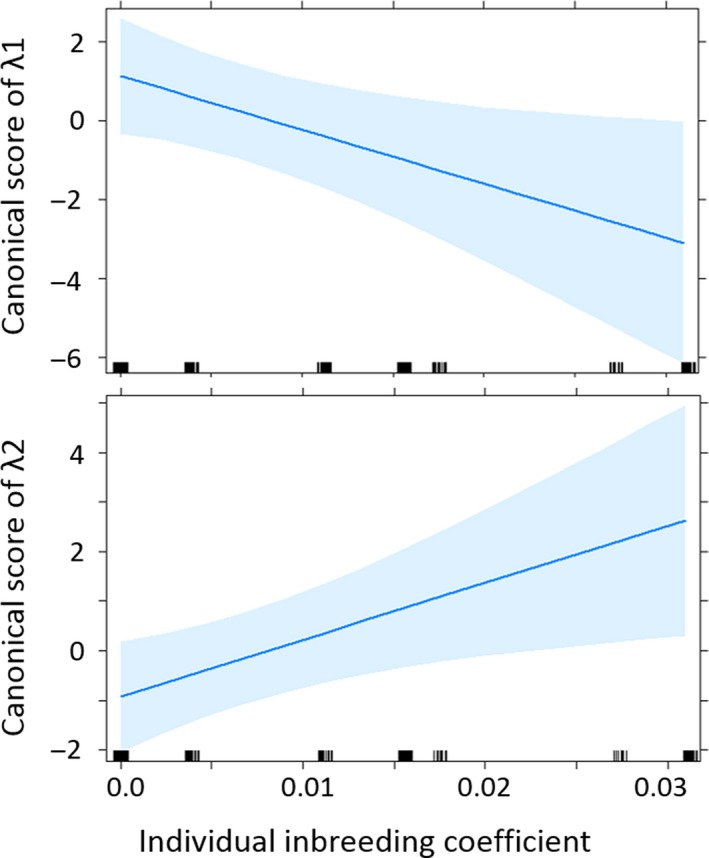
Relationships between the λ1 discriminant function and the λ2 discriminant function of the DFA and the inbreeding coefficient of 36 houbara bustard captive males. On each panel, the line shows the regression from a mixed effects linear model and the shaded band shows 95% confidence interval

The discriminant functions λ1 and λ2 were both significantly related to inbreeding coefficient (Figure 2). The inbreeding coefficient was negatively related to λ1 (slope = −136.5 ± 59.7, *p* = 0.029) and positively related to λ2 (slope = 114.9 ± 45.6, *p* = 0.017). As highest values of λ1 correspond to the lowest frequency booms, the males with the highest inbreeding coefficients produced the highest frequency booms (Figure 2). The relationships between each acoustic parameter and the inbreeding coefficient are presented in Supporting Information Figure S1 (see detailed results in Supporting Information Table S2). These results confirm that the frequency of booms was positively correlated with individual inbreeding (H1s, *p* = 0.009; H1e, *p* = 0.02).

### 3.3 Spatial structure of acoustic signals in the wild

The DFA including the 11 parameters measured on booms of the males in the wild identified 11 significant linear functions that allowed the maximizing of individual segregation. The DFA assigned 79.13% of the booms to the correct male and the cumulative percentage of the first two functions explained 80.25% of the total variance (lambda Wilks = 0.00109, *F*(176.14) = 88.26, *p* < 0.001; Table 2). There was no correlation between the spatial distance matrix and the acoustic distance matrix of wild males (simple Mantel test: *r* = −0.055, *p* = 0.531), indicating no spatial structure of acoustic signal (booms) within the population of males recorded.

**Table 2:** The first two discriminant functions of the discriminant function analysis performed with 11 acoustic parameters of the booms produced by wild males (explaining 80.248% of the total variance)

Axis	λ1	λ2
BD	0.115	0.225
Q25	−0.219	0.169
Q50	**−0.744**	0.281
Q75	−0.001	**0.95**
*E* < 100 Hz	0.492	−0.25
F0	−0.063	0.029
H1	**−0.808**	0.038
H2	**−0.728**	−0.082
H1s	**−0.596**	0.083
H1e	**−0.642**	−0.002
FM	0.044	−0.015
PEV (%)	53.04	27.208
RD (%)	79.127	

Note

PEV: proportion of variance explained; RD: discrimination rate.

Measures that contributed the most to the two first functions are in bold. Abbreviations and definitions of parameters are provided in the Section 2.2.4.

## 4 DISCUSSION

### 4.1 Relationship between booms parameters and genetic relatedness

We found a significant relationship between the kinship coefficients of captive houbara males and the frequency parameters, the frequency modulation and the duration of their booms, suggesting that several features of the booms are to some degree heritable through genetic transmission in this species. In other bird species, frequency and temporal acoustic parameters have been found to be genetically inherited as, for example, in the calls of northern bobwhites *Colinus virginianus* (Baker & Bailey, 1987) and in the begging calls of barn swallows *Hirundo rustica* and cliff swallows *Petrochelidon pyrrhonota* (Medvin, Stoddard, & Beecher, 1992). In vertebrates, call structure similarity between related individuals is often the outcome of inherited morphological, physiological, and neurological characters involved in call production (Bradbury & Vehrencamp, 2011). For instance, in several mammal species a negative correlation has been found between inherited traits such as body size or mass and the fundamental frequency or the formant frequencies of the vocalizations (Maynard‐Smith & Harper, 2003; Mousseau & Roff, 1987; Taylor & Reby, 2010). In birds, such covariation between body mass and frequency parameters of nonlearned vocalizations has been shown in the zebra finch *Taeniopygia guttata*, whereas learned male song traits such as repertoire size, motif length, or syllable rate showed poor heritability (Forstmeier et al., 2009). Male houbara bustards do not learn their vocalizations, and boom parameters appear to be transmitted through strict genetic inheritance and seem also to be linked to phenotypic constraints since they are related to the body weight (Cornec et al., 2015), which is also heritable in this species (Chargé et al., 2013).

### 4.2 Kin signaling and breeding strategy of houbara males

Heritable indicators of kinship may play an important role in some lekking species where kin selection (recognition and association) has been suggested to be involved both in the evolution and in the maintenance of the lek mating system (Kokko & Lindström, 1996; Sherman, 1999). In such species, kin selection may be favoured by strong reproductive skew between males and mutual benefits (direct and indirect) for related dominant and low‐ranked males (Kokko & Lindström, 1996; Sæther, 2002) clustering in larger leks and being then more attractive for females (the “female preference model”; Bradbury, 1981). This mechanism has been proposed in several species to explain the spatial distribution of related males and *in fine* their lekking behavior, as in the peacock *Pavo cristatus *(Petrie et al., 1999), the wild turkey *Meleagris galapavo* (Krakauer, 2005), and the white‐bearded manakin *Manacus manacus* (Shorey et al., 2000 but see Höglund & Shorey, 2003). Kin association has also been presented as providing direct benefits for low‐ranked males through the increase of mating opportunities (sperm depletion, female mistakes, queuing for mating (death of related high‐ranked male), see Sæther, 2002).

Association between relatives within leks might also be simply the result of a strong philopatry in males as outlined in the lesser prairie‐chicken *Tympanuchus pallidicinctus* (Bouzat & Johnson, 2004), the black grouse *Tetrao tetrix* (Höglund, Alatalo, Lundberg, Rintamaki, & Lindell, 1999), and the capercaillie *Tetrao urogallus* (Regnaut, Christe, Chapuisat, & Fumagalli, 2006; Segelbacher, Wegge, Sivkov, & Höglund, 2007). In the houbara, the probability of young males to encounter kin in their dispersal process is likely, due to their limited dispersal distance (35 km in average; Hardouin et al., 2012) and their faithfulness to their displaying site over years (Hingrat et al., 2008). Nevertheless, in our study, no significant correlation was found between the acoustic parameters of booms and spatial distances between males in the wild. Because the size of our sample of males in the wild is relatively small (we recorded 252 sequences of booms in 17 males), we may have missed an existing moderate correlation due to the lack of statistical power. However, our analysis suggests an absence of strong correlation between vocalization and spatial location of males during the breeding season, thus an absence of strong spatial kin clustering, which agrees with previous genetic findings on the absence of relatedness among males within leks (Lesobre, 2008). Our results also suggest that no alternative mechanisms (such as response to common environmental conditions or phenotypic interactions among individuals within leks) shape similar acoustic parameters of booms among males of the same lek.

Other studies failed to find evidences for kin selection among lekking birds (DuVal, 2007; Gibson et al., 2005; Loiselle et al., 2006; Martín, Alonso, Alonso, Pitra, & Lieckfeldt, 2002; McDonald & Potts, 1994), highlighting that alternative processes can explain the evolution and the maintenance of lekking behavior (Beehler & Foster, 1988; Bradbury & Gibson, 1983; Bradbury, Gibson, & Tsai, 1986; Höglund & Alatalo, 1995). In the houbara bustard, Lesobre et al. (2010) found no evidence for male reproductive skew within leks, as well as no preference for larger leks since males displaying solitarily or in leks have equal access to reproduction. Consequently, the “hotspot” remains a good candidate model to explain lek formation in the houbara (Hingrat et al., 2008), where males aggregate at highest female densities to increase their probability of mating (Bradbury et al., 1986). Besides its potential direct and indirect benefits for high‐ and low‐ranked males, such clustering can also be a constraint through the enhancement of male competition (Foster, 1983), even between close kin (Sæther, 2002) and through the decrease of per capita mating success of related males if female choice is based on traits that they equally share. Then, males may avoid their kin when clustering in order to reduce competition.

### 4.3 Inbreeding and acoustic signals

Interestingly, our results highlight that frequency parameters and the duration of the booms produced by males also reflect the level of individual inbreeding. Previous studies showed that individual heterozygosity or inbreeding is good predictors of the capability to acquire and hold territories (fur seals *Arctocephalus gazella*, Hoffman et al., 2007; wire‐tailed manakin *Pipra filicauda*, Ryder et al., 2010), the territorial size and status of males (black grouse *Tetrao tetrix,* Höglund et al., 2002; subdesert mesite *Monias benschi,* Seddon et al., 2004), their aggressive behavior (house mice *Mus domesticus,* Eklund, 1996) or their dominance (fur seals *Arctocephalus gazella,* Hoffman et al., 2007) and influences the expression of sexual traits (sedge warblers *Acrocephalus schoenobaenus*, Marshall, Buchanan, & Catchpole, 2003; song sparrows *Melospiza melodia*, Reid et al., 2005; zebra finches *Taeniopygia guttata*, Bolund, Martin, Kempenaers, & Forstmeier, 2010). In the houbara, frequency parameters of the booms have been demonstrated to support information exchanged during male–male interactions (Cornec et al., 2015). From these results, we can infer that inbred males producing high‐frequency booms should be disadvantaged in terms of competitiveness (agonistic relationships) and likely in their ability to acquire and hold a display site along the season and between years.

Moreover, if female choice relies on traits potentially affected by inbreeding, choosy females are expected to avoid inbred males (Hoffman et al., 2007; Höglund et al., 2002; Ryder et al., 2010; Sardell, Kempenaers, & Duval, 2014). Even though there is no post‐copulatory investment of the male in houbara reproduction, females might, by this way, avoid reproductive issues such as lower fecundity or hatching success which have been shown to be associated with inbreeding and homozygocity in many species (reviewed in Kempenaers, 2007). Moreover, inbreeding has been shown to have detrimental effects on individuals’ fitness by affecting growth rate, development stability, immuno‐competence, and survival (reviewed in Kempenaers, 2007). Therefore, females may increase offspring heterozygosity and fitness through kin recognition by mating with males carrying dissimilar alleles.

## 5 CONCLUSION

Among the diversity of mating strategies described in the last decades, the physiological and behavioral traits allowing individual to assess conspecific's genetic distance, compatibility, or quality remain poorly known. Here we provide evidence that acoustic signals, known as strong components of houbara bustard males’ courtship behavior, are related to both male identity and quality, making acoustic communication an effective channel to provide genetic information among conspecifics. Further studies, such as playback experiments, are needed to validate our hypotheses and assess the importance of the genetic information transferred by male vocalizations in shaping their intra‐ and intersexual relationship relationship.

## CONFLICT OF INTEREST

The authors declare that they have no conflict of interest.

## AUTHORS' CONTRIBUTIONS

C.C. designed the study, collected field data, analyzed data, and wrote the manuscript. A. R., F. R., and Y.H. collaborated in the conception and in the design of the study, coordinated the field operations of the study, and wrote the manuscript. All authors gave final approval for publication.

## ETHICAL STANDARDS

The experiments comply with the current laws of the country in which they were performed.

## Data Availability

The raw data supporting this research are openly available from the Dryad data archive at https://doi.org/10.5061/dryad.r8576v7.
