# Supplementary material for: Male vocalizations convey information on kinship and inbreeding in a lekking bird
Source: Ecol Evol. 2019 Mar 28;9(8):4421–30. doi: 10.1002/ece3.4986 (PMC6476769; doi:10.1002/ece3.4986)
Supplement: Supplementary file 1 [file ECE3-9-4421-s001.docx]

**Supplementary information for:**

**Male vocalizations convey information on kinship and inbreeding in a Lekking bird**

Cornec C.^1,2,^*, Robert A.^3^, Rybak F.^1^ & Hingrat Y.^4^

^1^ Institut des Neurosciences Paris-Saclay, Université Paris-Sud, CNRS (UMR 9197), 91405, Orsay, France

² Emirates Center for Wildlife Propagation, PoBox 47, Missour, Morocco,

^3^ Centre d'Ecologie et des Sciences de la Conservation (CESCO), Muséum national d'Histoire naturelle, Centre National de la Recherche Scientifique, Sorbonne-Université, 43 rue Buffon, 75005 Paris, France.

^4^ Reneco International Wildlife Consultants LLC., PoBox 61741, Abu Dhabi, United Arab Emirates.

* Corresponding author: clement.cornec@hotmail.fr

**Table S1**. Median repeatabilities (R) of acoustic parameters for the 36 captive males. Lower CI and upper CI are respectively the 2.5% and 97.5% percentiles and p-val is the p-value of the likelihood ratio test. BD: boom duration; F0, H1, H2: fundamental and harmonic frequencies; Q25, Q50, Q75: frequencies corresponding to 25, 50 and 75 % of the energy; E<100Hz: value of energy below 100 Hz; FM: frequency modulation; H1s: 1^st^ harmonic at the start of the boom; H1e: 1^st^ harmonic at the end of the boom.

|  | **R** | **lower CI** | **upper CI** | **p-val** |
| --- | --- | --- | --- | --- |
| **BD** | 0.737 | 0.62 | 0.817 | <10^-4^ |
| **Q25** | 0.563 | 0.42 | 0.675 | <10^-4^ |
| **Q50** | 0.753 | 0.648 | 0.831 | <10^-4^ |
| **Q75** | 0.344 | 0.221 | 0.467 | <10^-4^ |
| **E100HZ** | 0.827 | 0.737 | 0.882 | <10^-4^ |
| **F0** | 0.816 | 0.728 | 0.874 | <10^-4^ |
| **H1** | 0.902 | 0.848 | 0.936 | <10^-4^ |
| **H2** | 0.709 | 0.596 | 0.796 | <10^-4^ |
| **H1s** | 0.786 | 0.681 | 0.855 | <10^-4^ |
| **H1e** | 0.802 | 0.705 | 0.863 | <10^-4^ |
| **FM** | 0.837 | 0.74 | 0.894 | <10^-4^ |

| **Variable** | **Estimate** | **SE** | **df** | **T** | **p-value** |
| --- | --- | --- | --- | --- | --- |
| **BD** | -0.9 | 0.508 | 33.132 | -1.779 | 0.084 |
| **Q25** | -112.439 | 157.228 | 33.147 | -0.715 | 0.48 |
| **Q50** | 118.304 | 64.001 | 33.003 | 1.848 | 0.074 |
| **Q75** | 237.14 | 245.15 | 32.94 | 0.967 | 0.34 |
| **E<100Hz** | -2.371 | 1.473 | 33.004 | -1.61 | 0.117 |
| **F0** | 51.957 | 31.799 | 33.052 | 1.634 | 0.112 |
| **H1** | 127.243 | 67.174 | 33.011 | 1.894 | 0.067 |
| **H2** | 106.195 | 102.185 | 32.871 | 1.039 | 0.306 |
| **H1s** | 178.3 | 64.419 | 32.983 | 2.768 | 0.009 |
| **H1e** | 139.12 | 57.478 | 32.995 | 2.42 | 0.021 |
| **FM** | -525.64 | 297.005 | 33.044 | -1.77 | 0.086 |

**Table S2**. Relationships between individual inbreeding and 11 acoustic parameters based on linear mixed effects models. BD: boom duration; F0, H1, H2: fundamental and harmonic frequencies; Q25, Q50, Q75: frequencies corresponding to 25, 50 and 75 % of the energy; E<100Hz: value of energy below 100 Hz; FM: frequency modulation; H1s: 1^st^ harmonic at the start of the boom; H1e: 1^st^ harmonic at the end of the boom.

**Figure S1**. Relationships between acoustic parameters and the individual inbreeding coefficient of 36 houbara bustard captive males. On each panel, the line shows the regression from a mixed effects linear model and the shaded band shows 95% confidence interval. BD: boom duration; F0, H1, H2: fundamental and harmonic frequencies; Q25, Q50, Q75: frequencies corresponding to 25, 50 and 75 % of the energy; E<100Hz: value of energy below 100 Hz; FM: frequency modulation; H1s: 1^st^ harmonic at the start of the boom; H1e: 1^st^ harmonic at the end of the boom.

**
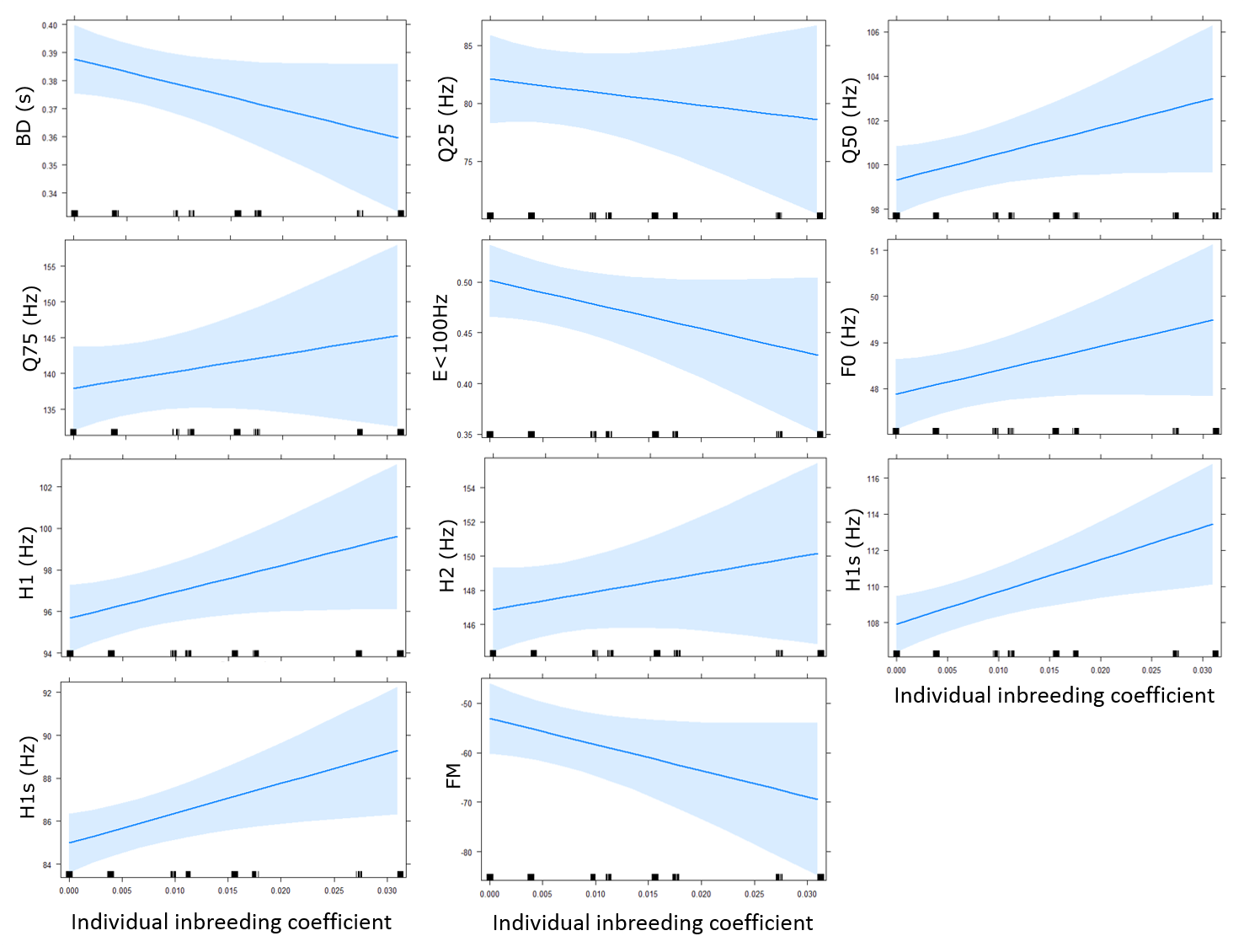
**
